# Upscaling human activity data: A statistical ecology approach

**DOI:** 10.1371/journal.pone.0253461

**Published:** 2021-07-01

**Authors:** Anna Tovo, Samuele Stivanello, Amos Maritan, Samir Suweis, Stefano Favaro, Marco Formentin

**Affiliations:** 1 Dipartimento di Fisica e Astronomia “Galileo Galilei”, Istituto Nazionale di Fisica Nucleare, Università degli Studi di Padova, Padova, Italy; 2 Dipartimento di Matematica “Tullio Levi-Civita”, Università degli Studi di Padova, Padova, Italy; 3 Padova Neuroscience Center, Università degli Studi di Padova, Padova, Italy; 4 Dipartimento di Scienze Economico-Sociali e Matematico-Statistiche”, Università degli Studi di Torino, Torino, Italy; Instituto de Fisica Interdisciplinar y Sistemas Complejos, SPAIN

## Abstract

Big data require new techniques to handle the information they come with. Here we consider four datasets (email communication, Twitter posts, Wikipedia articles and Gutenberg books) and propose a novel statistical framework to predict global statistics from random samples. More precisely, we infer the number of senders, hashtags and words of the whole dataset and how their abundances (i.e. the popularity of a hashtag) change through scales from a small sample of sent emails per sender, posts per hashtag and word occurrences. Our approach is grounded on statistical ecology as we map inference of human activities into the unseen species problem in biodiversity. Our findings may have applications to resource management in emails, collective attention monitoring in Twitter and language learning process in word databases.

## Introduction

In ecology one of the most studied emerging patterns is the *Relative Species Abundance* (RSA), that gives the fraction of species with the same number of individuals. To determine large scale RSA features from the distribution of species abundances within a small random sample is a major challenge in ecology and through years plenty of methods have been proposed [1–7]. The success of such methods depends on the following notable fact: different ecosystems like tropical forests or coral reefs, despite their disparate locations and different evolutionary history, share a common shape of the empirical RSA [7–10]. Recently in [8, 11], it has been developed a rigorous statistical framework to predict global scale biodiversity from scattered local plots. The core of the method is that the empirical RSA patterns can be well described by negative binomials, a distribution which has a theoretical foundation within the framework of neutral theory of ecology [9, 12]. In [8, 11], authors show that the mathematical properties of the negative binomial allow to estimate the number of species populating an ecosystem from knowing species abundances in a small portion of that ecosystem. The framework in [8, 11] also links RSAs to another informative pattern studied in theoretical ecology, the *Species Accumulation Curve* (SAC), which describes how the number of different species increases with the population size. In general, a log-series like RSA implies that the SAC is a logarithmic function of the population size [7, 10].

In the present paper we adopted and extended the statistical framework presented in [8, 11] and firstly designed in ecology to get new insights into human activity databases (i.e. email communication, Twitter posts, Wikipedia articles and Gutenberg books) with the aim of inferring global statistics of a dataset from a random sample of it. Statistical regularities have been widely observed in many different contexts of human dynamics—for instance, Zipf’s laws have been observed since decades in computational linguistic (see [13, 14] for a review)—and a variety of models have been proposed to explain such recurrent patterns [15–29]. In particular, the analogies between the systems here considered and ecological systems have been of interest for researchers and some modeling approaches move from an ecological perspective. For example, the analogy between the diffusion of online social media content and ecology has been investigated in [30–33]. The number of retweets of an hashtag may be interpreted as the offspring and one may think that the number of retweets depends on the attention a given hashtag is capable to gain. In this view, attention is the resource hashtags are competing for and lack of attention may bring to extinction. As far as the connection between words in books and ecological systems we can notice the following. Written language is (at least in first approximation) the account of spoken/natural language and like species, a language can split into several languages, it can mutate by modifying words/expressions over time, and it can face extinction. This connection has been explored intensively in the last decades. Many approaches to model language dynamics draw fully from ecology, for instance introducing the fitness of a language and competition between languages and words. A review of these research lines may be found in [34].

**Fig 1:**
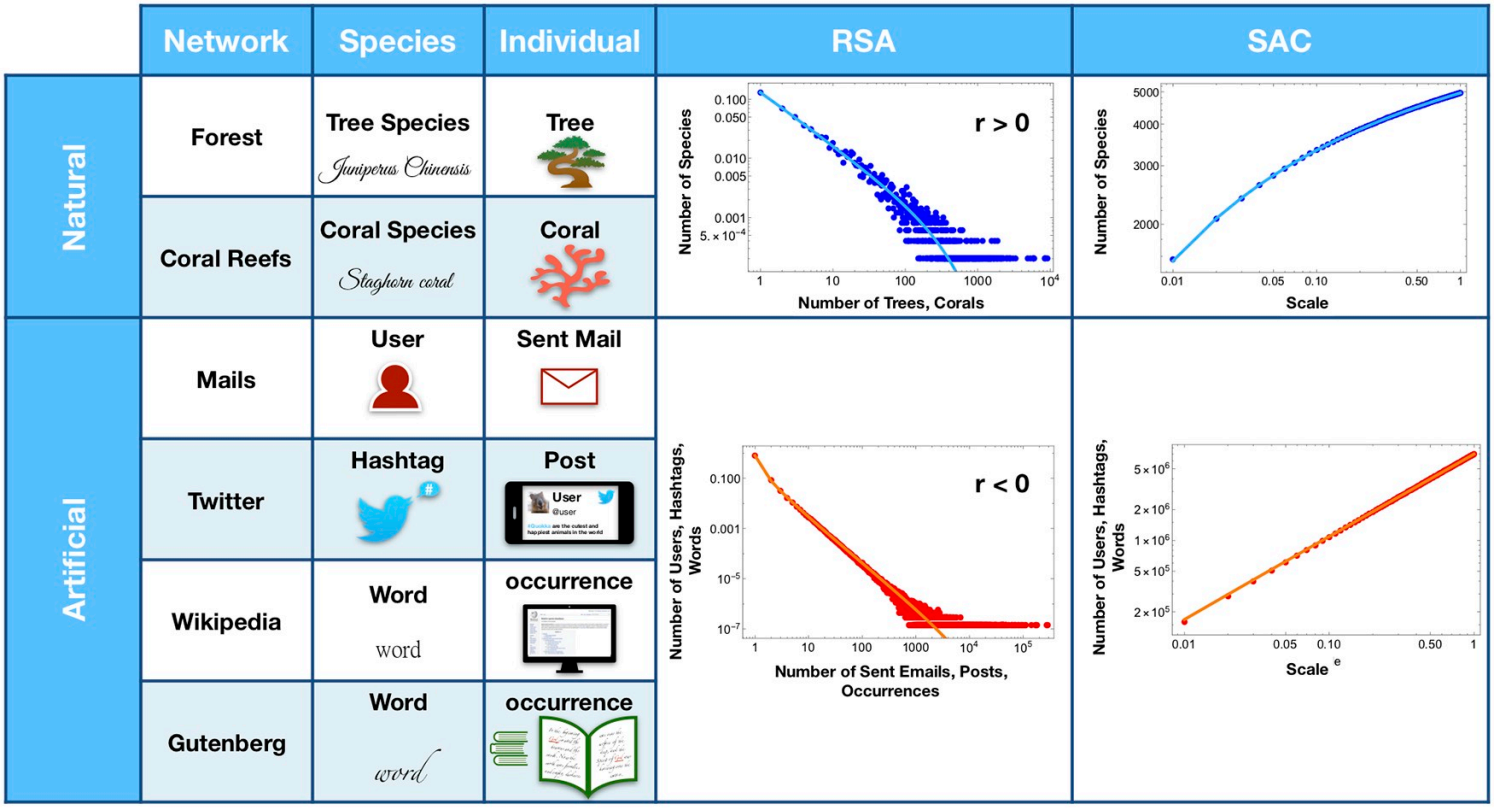
From ecology to human activities. The figure depicts the correspondence between species/individuals in a natural ecosystem and users/sent emails, hashtags/posts, words/occurrences in each one of the four datasets considered in the paper. Once the proper correspondence is established, it turns out that both natural and artificial RSAs can be well described by a negative binomial distribution. In the latest two columns, in order to show the typical shapes of the RSA (i.e. Relative Species Abundance) and SAC (i.e. Species Accumulation Curve) curves for natural versus human activity systems, we display the empirical patterns obtained for the Amazonia rainforest [8] and Twitter dataset. In general, all human activity RSA curves can be accommodated by with a negative value of clustering coefficient *r* in the interval (−1, 0) (see Eq 1), whereas natural ecosystems prefer *r* > 0 (solid lines).

**Fig 2:**
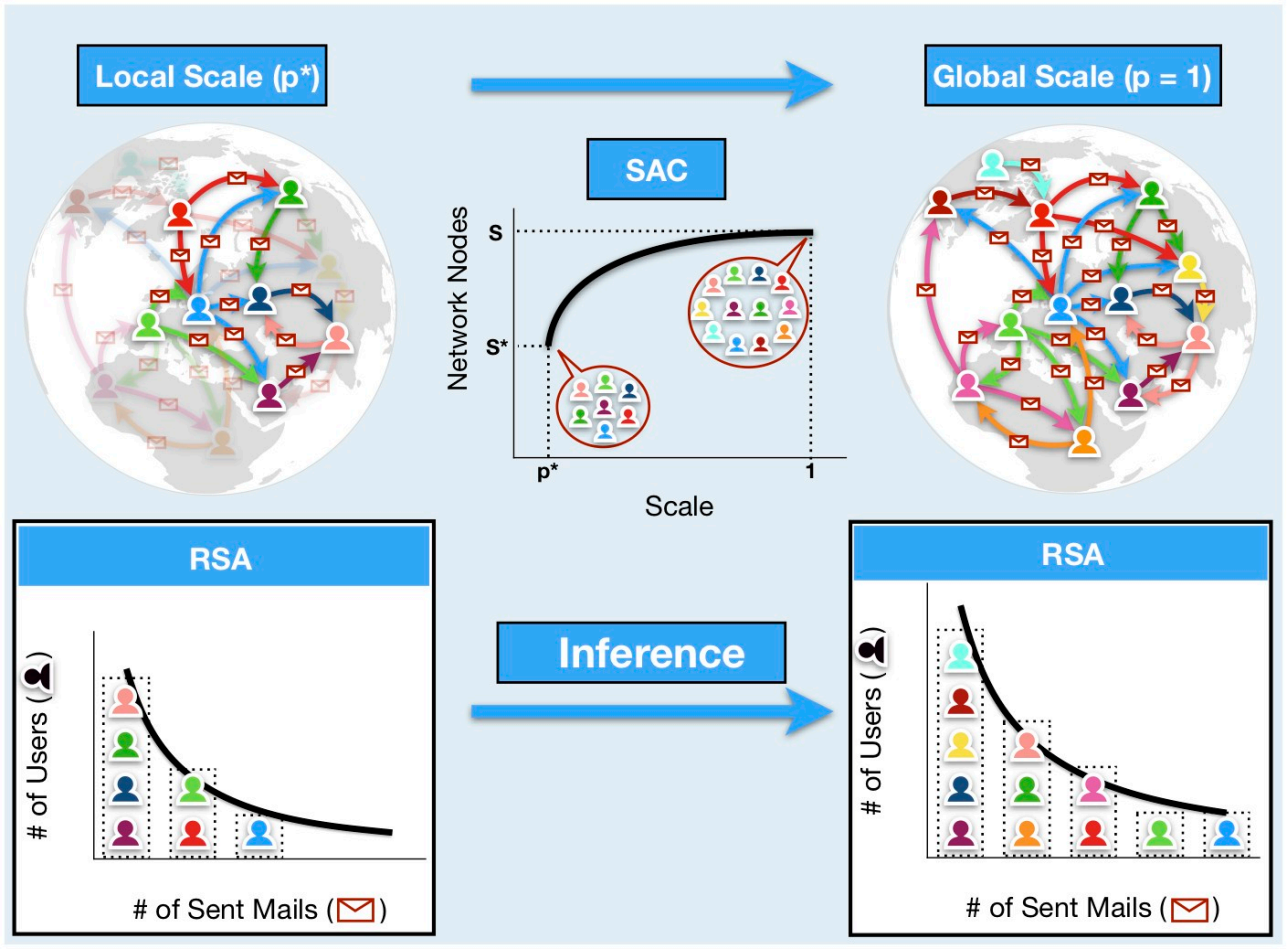
Sketch of our theoretical framework. Consider the email senders’ network where each node is a sender and a directed link from node *A* to node *B* is an email issued from user *A* to user *B*. We set the identity of a sender to be the species and a sent email to be an individual of that species. For instance, if the user *A* has sent *n* emails, then the species *A* has *n* individuals. If an observer has access to a fraction p* of the sent emails, s/he can partially recover the network (top-left) and the RSA curve at the local scale *p* (bottom-left). Within our framework, this information suffices to infer the number of species and the RSA curve at the global scale *p* = 1 (bottom-right). In terms of the network, the number of species corresponds to the number of users or nodes and the RSA gives the degree statistics. In this sense, our method reveals network features pertaining to the whole community activity initially unknown to the observer (top-right). Moreover, we can predict how the number of users increases with the number of links recorded, (i.e. the SAC curve in ecology), an information that may be used to optimize network design forecasting its growth.

In the present work we focus on inference, not modeling. In particular, we consider four human activities with the following correspondence between species and individuals within each dataset (see Fig 1): (1) *Email communication* [35, 36]: here we set the sender identity to label a species and the number of sent emails to be the number of individuals pertaining to a species; (2) *Twitter posts* [37]: here hashtags play the role of species and the number of different tweets containing a certain hashtag represents its population size; (3) For *Wikipedia articles* [37] and (4) *Gutenberg books* [37] we use the following setting: each word is a different species while its abundance is given by the number of occurrences of the word in the dataset. Once defined what corresponds to species and individuals, the RSA of each dataset displays a negative binomial behavior (see S1.1 Section in S1 Appendix). This permits to apply a statistical ecology approach to upscale human activity data. Within this framework, we propose a statistical method:

1. that gives reliable estimates for the number of users, hashtags, and words from a random sample of mails, posts and word occurrences. We refer to the inference of global quantities of interest from random samples as *upscaling* (see Fig 2). Moreover, our framework predicts how the number of users/hashtags/words grows with the recorded activity (mails/posts/pages/books): the rate at which new elements appear (i.e. the analogous of the SAC for human-activity data) shows a sublinear power-law growth, signature of the Heap’s law (see Fig 1).
2. We infer how the abundance of a species may change across scales (see Results section). This for example means that, observing a small portion of tweets and the popularity of a given hashtag among them, we can predict whether it will remain popular or not in the unseen part of the network.

In our statistical model we make the hypothesis the RSA distribution of the four human activity datasets can be described by a negative binomial with a clustering coefficient in the range (−1, 0) (see the results of Kolmogorov-Smirnov tests in S1 Table and S1.1 Section in S1 Appendix). This choice is justified by the heavy tail of the observed RSAs and the fact that the tail exponents are invariant under random sampling. Indeed, the negative binomial is (to our knowledge) the simplest *form-invariant* distribution. Form-invariance should not be confused with scale-invariance, a property only satisfied by power-laws (see S1.3 Section in S1 Appendix). With form-invariance we mean that when a portion of individuals are randomly sampled, the resulting RSA is still negative-binomially distributed with a heavy tail showing the same exponent as of the whole dataset (see S1.3 Section in S1 Appendix). Form-invariance property of the RSA allows us to build reliable estimators for the number of new features (new email users, new hashtags, new words) at each scale of the dataset starting from random samples of the whole databases. Our approach brings two main novelties/advantages. First, the choice to model the distribution of the occurrence frequencies according to a negative binomial distribution, supported by Kolmogorov-Smirnov tests (see S1.1 Section in S1 Appendix) is new. In particular, its form-invariance property allows us to obtain, for the quantities of interest, effective yet simple estimators which explicitly depend on the scale. In linguistic different parametric and non parametric statistical models has been used to infer how the number of types grows as new samples are added [13]. Instead, to our knowledge, upscaling has never been investigated for email communication and Twitter datasets. Second, within our framework we also derive an estimator for how the type abundances change across scales. This problem, as far as we know, has not been previously investigated although it could be of interest when interpreting abundances of types as a measure of popularity in social network data.

## Results

To start with, we illustrate our approach, its potentiality and the kind of results it can provide as applied to e-mail communication. We consider the senders activity network where each node is a user and a directed link from node *A* to node *B* represents an email issued from user *A* to user *B*. We set the identity of a sender to label the species and the number of sent emails to be the individuals pertaining to a species. Thus, for instance, if user *A* has sent *n* emails we say that species *A* has *n* individuals. Suppose an observer have access to a small sample of sent emails, or, equivalently, to partial information on links and nodes of the email communication network. Our approach allows to infer the number of nodes (i.e. the number of users) and the link statistics of the whole network, thus revealing features previously unknown to the observer (see Fig 2).

**Fig 3:**
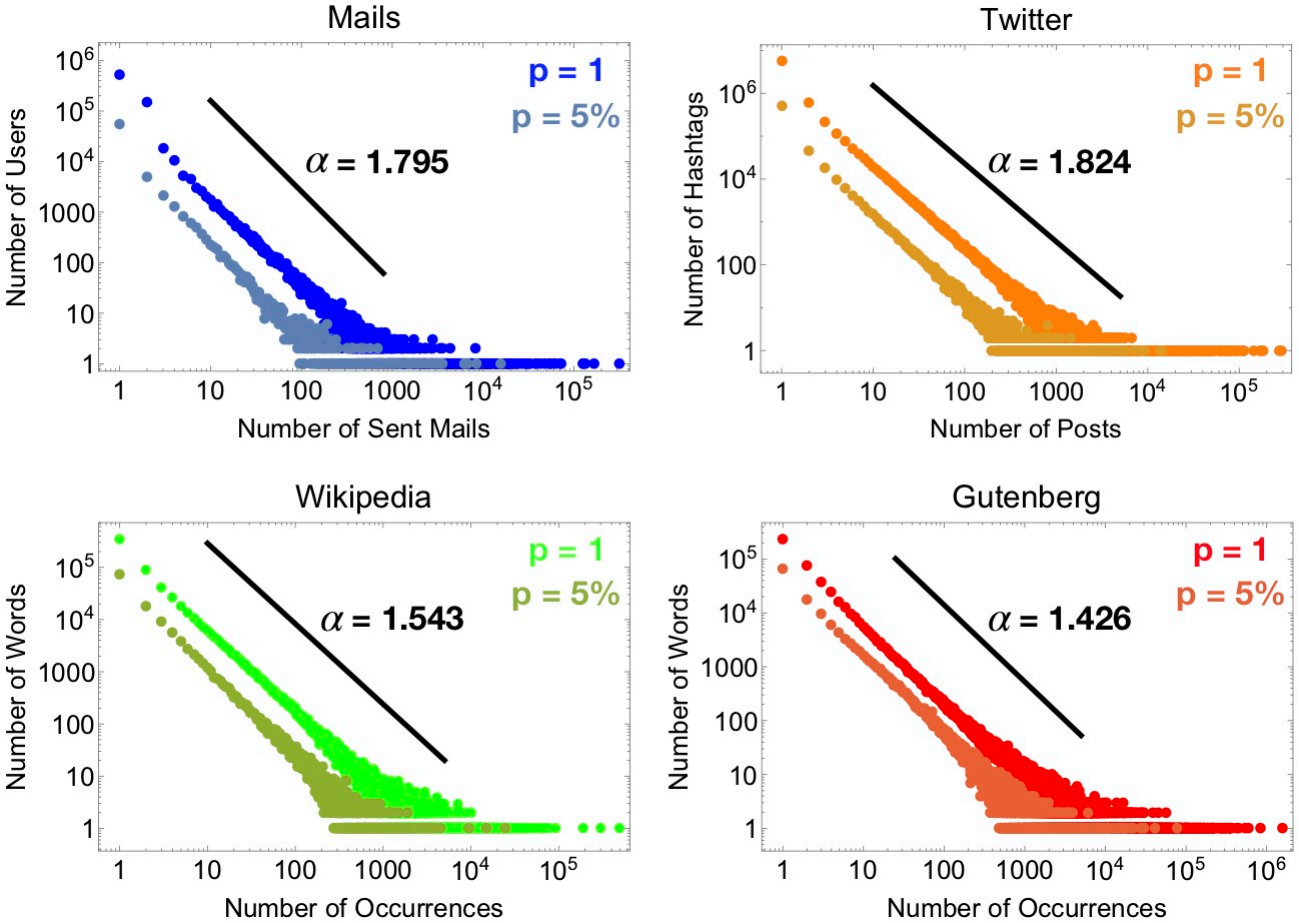
Universality and form-invariance of the empirical RSAs. Empirical RSA curves at the global scale (*p* = 1) and the local scale (*p* = 5%) are shown. The patterns show a heavy-tailed form maintained through the different human activities and scales. This scale-invariance property of the RSAs allows for a successful implementation of our theoretical framework. In particular, our model predicts that the heavy-tail exponent *α* is related to the clustering parameter *r* of the RSA negative binomial distribution via the relation *α* = 1 − *r* (see Materials and methods and S1.4 Section in S1 Appendix). In each plot, for a visual inspection, we inserted a black line with slope $-\alpha =-1+\hat{r}$, where $\hat{r}$ have been obtained by fitting the local patterns at *p* = 5% through a negative binomial (see also Table 1). We can see that such lines describe very well the heavy-tail regime of the empirical RSAs at both local and global scale in all four cases. For the RSA fitting curves and predicted patterns, see S1 Fig in S1 Appendix.

Correspondence between species/individuals and human activities can be set similarly for the remaining datasets (see Fig 1). Our statistical ecology approach gives the following results:

- **RSA universality and form-invariance**. In each activity the RSA of the whole dataset turns out to be heavy tailed with an exponent between -1.8 and -1.4 (see Fig 3). Moreover, this exponent is maintained at different scales (see S1 and S2 Figs in S1 Appendix), supporting our choice of modeling the RSA by means of a negative binomial, which is form-invariant and keeps fixed the tail exponent through scales.
- **Inference of unseen human activities**. On the scale invariance property of the RSA we build a statistical framework which gives robust and accurate estimates for the number of email senders, Twitter hashtags, words of Wikipedia pages and Gutenberg books from a random sample of sent mails, posts and word occurrences (see Table 1). Moreover, our framework allows to reconstruct the growth of the number of users/hashtags/words with the recorded activity (mails/posts/pages/books), which represents another well-known pattern in ecological theory called the *Species-Accumulation Curve* (SAC) (see Fig 2).
- **Popularity in social networks**. In Twitter and in social networks in general, popularity is known to be relevant, for instance, to manipulate mass opinion or to share information. One naive way to measure the popularity of a hashtag is to count the number of times it appears in other users’ tweets. In our ecological interpretation, a hashtag represents a species, while the number of posts associated to it, gives the species’ abundance. Within our framework, we can infer how the abundance of a species changes across scales (see Results section), thus allowing to monitor whether a locally popular hashtag will remain popular also in the undetected part of the network or not.

**Table 1:** Predicted relative errors. Upscaling results for the number of species of the four analysed datasets from local samples covering a fraction p* = 5% of the corresponding global dataset. For each human activity, we display the number of species (users, hashtags, words) and individuals (sent mails, posts, occurrences) at the global scale together with the average fitted RSA distribution parameters at the sampled scale and the relative percentage error (mean and standard deviation among 100 trials) between the true number of species and the one predicted by our framework. See S1 Fig in S1 Appendix for the corresponding fitting curves and predicted global RSA patterns.

	Emails	Twitter	Wikipedia	Gutenberg
Species	752, 299	6, 972, 453	673, 872	554, 193
Individuals	6, 914, 872	34, 696, 973	29, 606, 116	126, 289, 661
r	−0.796	−0.824	−0.543	−0.425
*ξ*_p*_	0.9999	0.9991	0.9985	0.9997
Relative Error	0.45 ± 0.45%	3.38 ± 0.16%	6.09 ± 0.38%	−2.44 ± 0.32%

In the following we give the key steps of our upscaling framework. Denote with *N* the population size and with *S* the number of species (i.e. senders, hashtags, words) of the whole database. Given a scale p* ∈ (0, 1), consider a random sample of size p* *N* in which we recover *S*_p*_ ≤ *S* species. In the sequel we denote by *P*(*n*|p*) the fraction of species with *n* individuals at scale p*, i.e. the sample RSA. We assume that, at the global scale *p* = 1, *P*(*n*|1) is proportional to a negative binomial distribution, P(n|r,ξ), with parameters *r* ∈ (−1, + ∞)\{0} and *ξ* ∈ (0, 1):

$$\begin{array}{c} P(n|1)=c(r,\xi )P(n|r,\xi )\;\text{for}\;n\geq 1 \end{array}$$ (1)

where the normalizing factor *c*(*r*, *ξ*) = 1/(1 − (1 − *ξ*)^*r*^) takes into account that each of the *S* species consists of at least one individual at the global scale.

RSAs given in (1) have the following features: 1) values of *r* ∈ (−1, 0) and *ξ* close to 1 reflect in a heavy-tailed behavior of the RSAs with an exponential cut-off. More precisely, the right tail of (1) has the form *n*^*r*−1^ exp(*n* log *ξ*) (see S1.4 Section in S1 Appendix), where the exponential cut-off is “small” for *ξ* close to one and disappears in the limit *ξ* → 1, where distribution (1) describes a pure power-law. Such heavy-tailed behavior with an exponential cut-off well describes the observed RSA patterns in human activities (see S1 Fig and S1.1 Section in S1 Appendix). Moreover, the exponent *α* = 1 − *r* matches very well with the empirical data (see also Fig 3).

2) Distribution (1) is *form-invariant*, meaning that the RSA *P*(*n*|*p*) maintains the same functional form at different scales *p* (see S1.3 Section in S1 Appendix), a property observed in the empirical RSAs of all the four databases (see Fig 3). In mathematical terms, the RSA at any scale *p* is again proportional to a negative binomial distribution with the same *r* and a rescaled parameter

$$\begin{array}{c} \xi_{p}=p\xi /\left(1-\xi \left(1-p\right)\right). \end{array}$$ (2)

Properties 1) and 2) are the building blocks of our predictive statistical framework.

Our goal is to infer the total amount of species *S* (senders, hashtags, words) present in the entire database given the number of species *S*_p*_ observed in a sample at the local scale p* and their corresponding abundance (number of mails, posts, occurrences). From this limited information, we can construct the empirical values of the RSA, *P*(*n*|p*), and fit it to obtain the estimates $\hat{r}$ and ${\hat{\xi }}_{p^{*}}$ of the parameters that best capture the behavior of the sampled data. Finally, thanks to the form-invariance property, one can obtain the value of the global parameter $\hat{\xi }$ via Eq (2) (henceforth we will denote with $\hat{\cdot }$ our estimation of any quantity ⋅).

Let us observe that the probability that a given species present at *p* = 1 is missing at *p* < 1 corresponds to the fraction of unobserved species (*S* − *S*_*p*_)/*S*. This value must be equal to *P*(0|*p*) = 1 − *c*(*r*, *ξ*)/*c*(*r*, *ξ*_*p*_), the probability for a species to have zero population in a sample of size *pN* (see S1.5 Section in S1 Appendix). Thus:

$$\begin{array}{c} \hat{S}\;≃\frac{S_{p^{*}}}{1-P(0|p^{*})}\;≃\;\frac{1-{\left(1-\hat{\xi }\right)}^{\hat{r}}}{1-{\left(1-{\hat{\xi }}_{p^{*}}\right)}^{\hat{r}}}S_{p^{*}}, \end{array}$$ (3)

where the last approximation is obtained by the definition of *c*(*r*, *ξ*) and expressing $\hat{\xi }$ as a function of ${\hat{\xi }}_{p^{*}}$ by inverting Eq (2).

To test the reliability of estimator (3), we extracted, from each dataset, a hundred sub-samples each covering a fraction p* = 5% of the databases’ individuals (sent emails, posted hashtags, occurrences of words). We then inferred the total number of species (email senders, posted hashtags in Twitter data and different words in Wikipedia pages and Gutenberg books) from the empirical RSA constructed at p* = 5%. The average relative upscaling error is small in all four cases: about 0.4% for sent emails, 3% for Twitter hashtags, 6% for Wikipedia words and -2% for Gutenberg words. In Table 1 we report the average values of the fitted parameters together with the average relative percentage error between the predicted number of species, $\hat{S}$, and the true one, *S* (mean and standard deviation are displayed for all datasets). See S3 Table and S3, S4 Figs in S1 Appendix for the results obtained by considering different fractions p* of the four datasets as starting information.

The second novelty that we introduce in our work is a method to estimate the variation of popularity, a fundamental concept arising naturally when investigating human dynamics [38–43]. Indeed, until now we exploited the information on the abundance of the observed species at the local scale only to estimate the number of unseen species, disregarding of their abundances. Instead, abundance information can be used to predict, for example, the most active users of the email network, the commonest words in a book or the popularity of a hashtag in Twitter database. In particular, focusing on Twitter, various sophisticated measures of popularity based on semantic analyses have been proposed (see for instance [44]). Here, by mean of the popularity of a hashtag we naively count the number of posts containing it that come to circulate within the network thanks to other users’ tweets. This information is encompassed within the RSA pattern. Indeed, hashtags posted a low number of times are those positioned in the left side of the curve, whereas hashtags with high popularity are located in its right tail. Our goal now is to derive an estimator for the change in popularity of hashtags from a portion p* of the observed tweets to the remaining 1 − p* fraction of the unobserved tweets.

Let us thus denote by *L* a fixed threshold of posts above which we consider a hashtag popular at the sampled scale p* and let us indicate with *S*_p*_(≥*L*) the number of different hashtags having abundance at least *L* in the surveyed collection of posts. We wish to check whether these (locally) popular species result to be popular also in the unseen fraction of the network, 1 − p*. Let us then denote by *K* the fixed popularity threshold at the unsurveyed scale. We are looking for an estimator of the number of species having popularity at least *K* in the 1 − p* unseen part of the tweets, given that they have popularity at least *L* at scale p*. These species, which we denote with ${\hat{S}}_{1-p^{*}}\left(\geq K|\geq L\right)$ are therefore globally popular within the network.

From our theoretical framework, we derive an estimator of such a quantity (see S1.6 Section in S1 Appendix). We define *S*_p*_(*l*) to be the number of species having popularity exactly *l* at scale p* and *S*_1−p*_(*k*|*l*) to be the number of species having popularity exactly *k* at scale 1 − p* given that they have popularity exactly *l* at scale p*. Then we obtained the following estimator for *S*_1−p*_(*k*|*l*) (see S1.6 Section in S1 Appendix for details):

S^1-p*(k|l)=Sp*(l)·(k+ll)p*l(1-p*)k(k+l+r^-1k+l)ξ^k+l(1-ξ^)r^(l+r^-1l)ξ^p*l(1-ξ^p*)r^=Sp*(l)(k+l+r^-1k)·p*l(1-p*)kξ^k+l(1-ξ^)r^ξ^p*l(1-ξ^p*)r^ (4)

An estimator for ${\hat{S}}_{1-p^{*}}\left(\geq K|\geq L\right)$ can thus be obtained by summing up (4) for all *k* ≥ *K* and for all *l* ≥ *L*. We tested the above estimator by fixing the (arbitrary) value of the threshold *L* equal to 25 and varying the value of *K* in the (arbitrary) range from 219 to 548 for ten sub-samples of Twitter database (for different choices of *L* and *K* see S4 Table in S1 Appendix). The average errors obtained in the predictions are displayed in Table 2. For all the considered cases, we achieved very good estimates, with an average relative percentage error below 0.2% in absolute value.

**Table 2:** Percentage errors for popularity change predictions in Twitter database. For a fixed *L* = 25 and different values of *K* (first and second column), we estimated, from ten different Twitter sub-samples (p* = 5%), the number of species having abundance at least *K* at the unobserved scale 1 − p* = 95% given that they have abundance at least *L* at the sampled scale p* via estimator (4). The average among the ten sub-samples of the true numbers of species, *S*_1−p*_(≥*K*|≥*L*), and of the ones predicted by our method, ${\hat{S}}_{1-p^{*}}\left(\geq K|\geq L\right)$, among the ten sub-samples are displayed in the third and fourth columns, respectively. Finally, in the last two columns, we inserted the mean and the variance of the relative error obtained in the ten predictions. Similar results have been obtained for other values of *L* and *K* (see S4 Table in S1 Appendix).

*L*	*K*	*S*_1−p*_(≥*K*|≥*L*)	${\hat{S}}_{1-p^{*}}\left(\geq K|\geq L\right)$	Relative Error	Variance
25	219	5, 977	5, 976.80	0.0018131	0.0000282
25	329	5, 943	5, 950.31	0.0448228	0.01097890
25	439	5, 667	5, 688.88	0.0896268	0.0609518
25	548	5, 064	5, 055.71	−0.1793290	0.0877951

## Discussions

To conclude, we show how our statistical ecology framework could be successfully applied to human activities. We tested our method in four databases: email sender activity, Twitter hashtags, words in Wikipedia pages and Gutenberg books. Once set the correspondence to what we consider species and individuals of a species, our approach reveals that the RSA is scale-free in each mentioned dataset with a heavy-tailed form maintained at different scales—with roughly the same exponent—through the different human activities considered (see Fig 3). This form-invariant property allows for a successful implementation of our predictive statistical framework. The importance of the form-invariance has been already noticed in network science; in [45] authors state that “Only if the degree distributions of the network and randomly sampled subnets belong to the same family of probability distributions is it possible to extrapolate from subnet data to properties of the global network.” In our language and framework this translates into “upscaling is possible when the distribution is form invariant”. However, the heavy tail of the observed RSAs cannot be captured by a standard negative binomial distribution with $r\in R^{+}$. Nevertheless, such behaviours can be accommodated when allowing the clustering parameter *r* to take negative values, *r* ∈ (−1, 0) (see Materials and methods, S1 Fig and S1.1 Section in S1 Appendix). This allows us to exploit the form-invariance property of the negative binomial distribution to propose an estimator for the statistics of the unseen human activity from small random samples. In particular, from the activity (sent emails per senders, posts per hashtags, word occurrences) in a small random sample, we infer the number of species (senders, hashtags, words) at the global scale. Moreover, we predict how the popularity of species changes with the scale, an issue of evident importance when thinking of social networks like Twitter. Finally, we compare our estimates with the true known values and in all the considered databases the relative error is small (see Tables 1, 2 and S3 Section in S1 Appendix). This result confirms the ability of our theoretical method to capture hidden quantities of the human dynamics when only random samples are available. In this regard, we remark that within our approach what matters is the ratio between the size (in terms of the number of items) of the random sample and the size of the target one. This implies that our statistical model allows to upscale to two, three, *n* times the size of the given sample. This feature may be useful when the size of the whole dataset is unknown. Our results pave the way for new applications in upscaling problems beyond statistical ecology.

Indeed, our findings may have applications in different situations, spreading from resource management in emails to collective attention monitoring in Twitter and to language learning process in word databases. Let us see one example for each aforementioned context of how our framework could help in decision making processes related to different aspects of social activity networks. Let us start from the resource managing application. Suppose an internet/email provider starts a campaign to increase customers; for instance the provider wishes to double the number of subscribers. Now, in order to predict if more resources (e.g. number of servers in the email example) are necessary to supply the newly entered subscribers, the provider needs to infer the total amount of activity bursting thanks to these new users. Our method provides a possible solution to this inference problem. Indeed, by inverting Eq (3), which represents the well-known species-accumulation curve in theoretical ecology, one obtains an analytical link between the total amount of activity (e.g. number of sent emails) and the number of users. In particular, the activity does not grow linearly with the users, as one may naively guess. Thus, the information our framework provides on the species-accumulation curve may help the provider to decide how many further resources are needed for the expected number of new users. Clearly, this knowledge is useful either to avoid money waste in case no further resources are required, or to provide new structures/servers in advance in order to safely support the user activity and not to loose unsatisfied customers. Moreover, being aware of how many new structures are needed also helps balance their costs of installation, managing and maintenance with the profit coming from subscriptions.

A second application regards attention monitoring and information spreading. Nowadays social networks constitutes a fundamental source for spreading information and disinformation as well. They have being exploited to influence the mass opinion and attention in many different social contexts, from politics to economy [46]. It is enough to think about the influencer phenomenon arising in almost all social networks. In Twitter, popularity of a user may be read from the number of times a hashtag s/he initiated appears in other users’ tweets. In our ecological interpretation, a hashtag represents a species, while the number of posts associated to it gives the species’ abundance. Therefore, if the species s/he represents comes to be part of the right tail of the RSA distribution, it constitutes one of the community dominant species and thus we can say s/he is popular, whereas if it comes to fall at the left tail of the RSA, it is a hyper-rare species, thus not having received the desired attention. Therefore, in order to control someone’s position within the global network, it is necessary to have access to the RSA at the whole community scale. However, this datum is usually not provided by the social network managing organization. Twitter, for example, only releases information on the total number of tweets posted across time. Nevertheless, there are other services as the Sample Tweets APIs or the Decahose stream service which provide the clients with real-time random samples covering small percentages (up to 10%) of the total tweets. With this information, our framework offers the possibility to fully reconstruct the global RSA as well as to monitor how the number of popular hashtags scales from the monitored sample up to the whole activity network. This latter information may also be useful for governments or public administrations in general to communicate important news (health information, emergency procedures, elections etc…) to the citizens. In particular, our method allows to know the number of further tweets one eventually needs to effectively spread the information, thus allowing to undertake the proper measures (a bigger publicity campaign to obtain more followers, the development of bot applications, etc.) to achieve the goal.

Finally, our theoretical framework may also be exploited in language learning process monitoring. For example, let us suppose one is learning a foreign speech. S/he may then be interested in the number of books that are needed s/he needs to read in order to be sure to expand her/his own vocabulary in order for it to cover a fixed percentage of all the speech words. The species-accumulation curve emerging in this context thanks to our ecological correspondence between words/species and occurrences/abundances can thus be interpreted in a broader sense as a learning curve, with the total number of words encountered during the learning process (by dialogue experience, frontal lectures or personal readings) in the x-axis and the number of different words s/he manages to properly exploit in her/his speech in the y-axis.

## Materials and methods

### Datasets

In this study we considered four databases concerning human activities: emails, Twitter, Wikipedia and Gutenberg. Here we give a brief description of the data. For further details, see [35] for email dataset and [37] for Twitter, Wikipedia and Gutenberg data.

#### Emails

This dataset is a collection of almost 7 millions emails, that corresponds to the activity of a Department of the Università degli Studi di Padova during two years: 2012 and 2013. The collected data are in the form {sender, receiver, timestamp}. For our analysis, we selected the first column of the table [35].

#### Twitter

Our dataset consists of a table where each row is of the form {timestamp, hashtag, user}. For our purposes, we selected the second column of the table. Dataset can be found in http://kreyon.net/waves-of-novelties/ [37].

#### Wikipedia and Gutenberg

Our data represents all words contained in a collection of Wikipedia pages and books. We label each different word with a different number. Note that the same word always maintain its correspondence to the same number, regardless of the Wikipedia page or book it belongs [37].

### Power-law tails of the negative binomial with a negative clustering coefficient

A negative binomial density function with parameters *ξ* and *r* > 0 results to capture very well empirical RSA patterns in tropical forests [8, 11]. The observed RSAs in the analyzed human-activity databases, although displaying a similar universal character, do show a different behavior, characterized by heavy tails (see Fig 3). These heavy tails of the observed RSAs cannot be captured by a standard negative binomial distribution with $r\in R^{+}$. Nevertheless, extending the clustering parameter region to take negative values, *r* ∈ (−1, 0), reflects in a power-law behavior of the RSA distribution tail with an exponential cut-off. To show this, let us consider a truncated negative binomial distribution of parameters *r* and *ξ* at the global scale (henceforth we will write *P*(*n*) for *P*(*n*|1)). The following theorem holds true [47, 48].

**Theorem**. *Let Y(z) be the generating function of a discrete random variable having probability mass function P*(⋅) *with dominant singularity R*_*Y*_. *Let* β∈R\{0,1,2,...}. *If for z* → *R*_*Y*_

$$\begin{array}{c} Y\left(z\right)∼c_{Y}{\left(1-z/R_{Y}\right)}^{\beta }, \end{array}$$

*then the distribution P*(*n*) *satisfies*

$$\begin{array}{c} P\left(n\right)∼\frac{c_{Y}n^{-\beta -1}R_{Y}^{-n}}{\Gamma (-\beta )}\;\;\text{for}\;n\to \infty , \end{array}$$

*where* Γ(⋅) *is the Gamma function*.

Let us thus examine the probability generating function of our truncated negative binomial:

$$\begin{array}{c} Y\left(z\right)=\sum_{n=0}^{\infty }P\left(n\right)z^{n}, \end{array}$$

where *P*(*n*) is given in (1). Now, since we are interested in the singularities of *Y*(*z*), we can neglect the normalizing factor *c*(*r*, *ξ*). Moreover, as the tail of a truncated negative binomial is exactly the same of a standard negative binomial, here we simply disregard of the truncation and conduct the analysis for a standard negative binomial. It then turns out (see S1.4 Section in S1 Appendix for details) that *Y*(*z*) has a singularity at *z* = 1/*ξ* and that it can be expressed as:

$$\begin{array}{c} Y\left(z\right)=c_{Y}{\left(1-z\xi \right)}^{-r}=c_{Y}{\left(1-z/R_{Y}\right)}^{\beta }, \end{array}$$

where we set *β* = −*r* and $R_{Y}=\frac{1}{\xi }$. Thus, Theorem above provides a characterization of the tails of the (truncated) negative binomial:

$$\begin{array}{c} P\left(n\right)\;∼\;\frac{c_{Y}n^{r-1}\xi^{n}}{\Gamma (-\beta )}\;=\;\frac{c_{Y}n^{r-1}e^{n\ln(\xi )}}{\Gamma (-\beta )},\;n\gg 1. \end{array}$$

The cut-off thus depends on *ξ*. In particular, the power-law range is greater for values of *ξ* close to 1.

## Supporting information

S1 Appendix Theoretical framework and additional results and figures. (PDF)
