# Supplementary material for: Upscaling human activity data: A statistical ecology approach
Source: PLoS One. 2021 Jul 1;16(7):e0253461. doi: 10.1371/journal.pone.0253461 (PMC8248688; doi:10.1371/journal.pone.0253461)
Supplement: S1 Appendix — (PDF) [file pone.0253461.s001.pdf]

S1 Appendix

S1 Theoretical framework

S1.1 Kolmogorov-Smirnov tests on data

In this section we wish to apply Kolmogorov-Smirnov to test the reliability of our assumption that a negative binomial (NB) with negative clustering coefficient well describes the human-activity data we are studying. Let us remark that this is only hypothesis on which we built our statistical model and by no means the core of the manuscript. Indeed, our scope is not to find the best fat-tailed distribution to fit data, but instead to find a statistical model to give reliable estimates of the quantities we are interested to infer. From this perspective, our choice of modeling the frequency of frequencies with a NB is justified by their form-invariance which allows to mathematically obtain an effective yet simple estimator for the global number of types which explicitly depends on the scale.

We thus proceeded as follows: for each global dataset, we generated two random sub-samples covering a fraction of 3% and 5% of all the individuals (posts, e-mails, words) and we used maximum likelihood estimation to obtain the NB r and ξ parameters providing the best-fit of the local empirical RSA distribution (see Figure S2 and Figure 3 of the main text). We thus generated 5000 data from the fitted distribution and applied Kolmogorov-Smirnov to compare the original and the generated data. We repeated the procedure 100 times for both the 3% and 5% samples. In Tables S1 and S2 we show the statistics (average, standard deviation and range) of the 100 p-values obtained for all the four datasets, as well as the corresponding percentage of times that the null hypothesis that the two datasets follow the same distribution is accepted at 1% and 5% level. We then applied the very same procedure when fitting the original data with a Zipf' law, which have been observed and studied since decades in computational linguistic to describe type-token relationships. In Tables S1 and S2 we also reported the results of Kolmogorov-Smirnov for this assumption. As we can see, for all dataset, the NB resulted compatible with data in more than 90% of the cases, whereas the Zipf's law was mainly rejected in the Emails, Twitter and Gutenberg dataset, while being accepted in 60% circa of the cases for Wikipedia.

p-value	Emails		Twitter		Wikipedia		Gutenberg	
statistic	NB	Zipf	NB	Zipf	NB	Zipf	NB	Zipf
mean	0.88	~ 0	0.85	$1.1 \cdot 10^{-6}$	0.41	0.099	0.48	0.0088
sd	0.18	~ 0	0.22	$8.4 \cdot 10^{-6}$	0.29	0.17	0.27	0.016
min	0.26	0	0.13	~ 0	0.00027	$\sim 10^{-6}$	0.00030	$2.3 \cdot 10^{-6}$
max	1	~ 0	1	0.000083	$\sim$1	0.82	0.998	0.080
accepted at 1%	100	0	100	0	96	66	98	20
accepted at 5%	100	0	100	0	92	41	93	3

Table S1. Kolmogorv-Smirnov Tests. Results for Kolmogorov-Smirnov test on the sub-samples covering the 3% of the global number of individuals in the four different human-activity datasets (columns 2 to 5). We report the p-values statistics (average, standard deviation, minimal/maximal value and number of cases in which the null hypothesis that data are compatible with the fitted NB/Zipf's law distribution is accepted at 1% and 5% level) among the 100 trials for each sub-sample.

p-value	Emails		Twitter		Wikipedia		Gutenberg	
statistic	NB	Zipf	NB	Zipf	NB	Zipf	NB	Zipf
mean	0.63	~ 0	0.90	$1.3 \cdot 10^{-7}$	0.36	0.062	0.45	0.0033
sd	0.25	~ 0	0.16	$5.6 \cdot 10^{-7}$	0.26	0.093	0.29	0.0091
min	0.076	0	0.10	0	0.0011	$1.1 \cdot 10^{-6}$	0.004	~ 0
max	$\sim$1	~ 0	1	$4.8 \cdot 10^{-6}$	~ 1	0.45	0.98	0.066
accepted at 1%	100	0	100	0	98	60	99	6
accepted at 5%	100	0	100	0	91	35	92	2

Table S2. Kolmogorv-Smirnov Tests. Results for Kolmogorov-Smirnov test on the sub-samples covering the 5% of the global number of individuals in the four different human-activity datasets (columns 2 to 5). We report the p-values statistics (average, standard deviation, minimal/maximal value and number of cases in which the null hypothesis that data are compatible with the fitted NB/Zipf's law distribution is accepted at 1% and 5% level) among the 100 trials for each sub-sample.

S1.2 Statistical model

Once it has been defined what are species and individuals of a species in each of the four human activities considered, we can proceed in the explanation of our statistical model from an ecological perspective.

We denote with N the total population size and with S the number of different species populating an ecosystem.

The *species abundance distribution* (SAD) at a scale p depicts the number of species in a subpopulation of size pN having exactly n individuals. In the following we will quote as RSA the corresponding probability distribution, denoted by $P(n|p)$.

Let us now consider the whole system, i.e. the entire population. We assume that, at the global scale $p = 1$, the RSA distribution is proportional to a negative binomial with parameters r and ξ. It reads:

$$P(n|1) = c(r, \xi) \cdot \mathcal{P}(n|r, \xi) \qquad \text{for } n \geq 1 \tag{1}$$

where $\mathcal{P}(n|r, \xi)$ is the well known negative binomial density function with parameters r and ξ, i.e.

$$\mathcal{P}(n|r, \xi) = \binom{n + r - 1}{n} \xi^n (1 - \xi)^r$$

and where the normalizing factor $c(r, \xi)$ takes into account the fact that each of the existing S species at the global scale consists of at least one individual:

$$c(r, \xi) = \left[\sum_{n=1}^{\infty} \binom{n + r - 1}{n} \xi^n (1 - \xi)^r \right]^{-1} = \frac{1}{1 - (1 - \xi)^r}.$$

Through the paper we always consider the generalized negative binomial distribution where the binomial coefficient is expressed by means of Gamma functions, i.e. $\binom{n+r-1}{n} = \frac{\Gamma(n+r)}{\Gamma(n+1)\Gamma(r)}$.

The reason why we chose to model the RSA distribution with a negative binomial will be clear in few lines. For the moment, let us anticipate that the negative binomial has two properties that are essential for the development of our estimators: it is form-invariant (see Section S1.2) and, varying the values of ξ and r, it can describe very well different tail behaviors, from exponential to power-law (see Section S1.3).

S1.3 Form-invariance of the RSA distribution

Zooming at a sub-scale p, i.e. considering a subpopulation of size pN, we will recover $S_p \leq S$ species. Note that S_p may depend on which pN individuals we select. In other

words, different samples of the same size may lead to different values of S_p. We wish to derive the distribution of the local RSA $P(k|p)$ under the hypothesis of random sampling.

Under random sampling, it can be proven that, if the RSA at the global scale is distributed according to (1), then the local RSA at a local scale p is again proportional to a negative binomial, with rescaled parameter ξ_p and same r:

$$P(k|p) = \begin{cases} c(r,\xi) \cdot \mathcal{P}(k|r,\xi_p) & k \geq 1 \\ 1 - c(r,\xi)/c(r,\xi_p) & k = 0 \end{cases} \tag{2}$$

with

$$\xi_p = \frac{p\xi}{1-\xi(1-p)}. \tag{3}$$

The fact that the RSA maintains the same functional form at different scales will be central in our framework. We will refer to this property as *form-invariance*. We remark that form-invariance should not be confused with *scale-invariance*. In fact, this latter is defined as the following property: a distribution f is said to be scale-invariant if $f(px) = g(p)f(x)$ where $g(p)$ is a multiplicative scale-dependent constant. It can be proven that power-laws are the only distributions satisfying this property. In contrast, with form-invariant we mean a distribution which maintains the same functional form under random sampling.

We wish now to prove that relation (2) holds.

Suppose that a species consists of n individuals among the whole population. Under random sampling, the conditional probability that the species has k individuals at the sub-scale p, given that it has total abundance n at the global scale, is given by a binomial distribution of parameters n and p:

$$\mathcal{P}_{binom}(k|n,p) = \binom{n}{k} p^k (1-p)^{n-k} \qquad k = 0, ..., n$$

and $\mathcal{P}_{binom}(k|n,p) = 0$ if $k > n$. Let us now prove that the RSA at the local scale $P(k|p)$ is indeed distributed according to (2).

We start by noticing that, in order to compute the probability that a species has abundance $k \geq 1$ at a local scale p, we need to condition on the fact that the species has abundance n at the whole scale $p = 1$, and then to sum over n, i.e.

$$\begin{aligned} P(k|p) &= \sum_{n \geq k} \mathcal{P}_{binom}(k|n,p) P(n|1) \\ &= \sum_{n \geq k} \binom{n}{k} p^k (1-p)^{n-k} \cdot c(\xi,r) \binom{n+r-1}{n} \xi^n (1-\xi)^r \\ &= c(\xi,r) \binom{k+r-1}{k} \left(\frac{p\xi}{1-\xi(1-p)}\right)^k \left(\frac{1-\xi}{1-\xi(1-p)}\right)^r \\ &= c(\xi,r) \binom{k+r-1}{k} \xi_p^k (1-\xi_p)^r \\ &= c(\xi,r) \cdot \mathcal{P}(k|r,\xi_p), \end{aligned}$$

with ξ_p given in (3). For $k = 0$ we have

$$P(0|p) = 1 - \sum_{k \geq 1} \mathcal{P}_{sub}(k|p) = 1 - c(\xi,r) \sum_{k \geq 1} \mathcal{P}(k|r,\xi_p) = 1 - \frac{c(\xi,r)}{c(\xi_p,r)}.$$

Our method proceeds as follows: after fitting the parameters $\hat{\xi}_{p^*}$ and $\hat{r}$ from the empirical RSA observed at a local scale p^*, we upscale them so to obtain an estimation of the global parameter $\hat{\xi}$ at $p = 1$ by inverting (3). The formula reads explicitly:

$$\xi = \frac{\xi_{p^*}}{p^* + \xi_{p^*}(1 - p^*)}. \tag{4}$$

Note that this form-invariance holds between any two scales $q \leq p$. Indeed, from

$$\xi_p = \frac{p\xi}{1 - \xi(1-p)} \qquad \text{and} \qquad \xi_q = \frac{q\xi}{1 - \xi(1-q)}$$

we obtain

$$\begin{aligned} \xi_q &= \frac{q\xi}{1-\xi(1-q)} = \frac{q\frac{\xi_p}{p+\xi_p(1-p)}}{1 - \frac{\xi_p}{p+\xi_p(1-p)}(1-q)} = \frac{q\xi_p}{p + \xi_p(1-p) - \xi_p(1-q)} \\ &= \frac{q\xi_p}{p - \xi_p(p-q)} = \frac{\frac{q}{p}\xi_p}{1 - \xi_p(1-\frac{q}{p})}. \end{aligned}$$

With the same argument, for any $q \geq p$ it holds

$$\xi_q = \frac{\xi_p}{\frac{p}{q} + \xi_p(1 - \frac{p}{q})}. \tag{5}$$

Hence what really matters is the relative ratio of the two scales.

S1.4 Power-law tails of $\mathcal{P}(n|r,\xi)$ with $r \in (-1, 0)$

A negative binomial density function with parameters ξ and $r > 0$ results to capture very well empirical RSA patterns in tropical forests [Tovo et al.(2017)Tovo, Suweis, Formentin, Favretti, Volkov, Banavar et al., Tovo et al.(2019)Tovo, Formentin, Suweis, Stivanello, Azaele, and Maritan]. The observed RSAs in the analyzed human-activity databases, although displaying a similar universal character, do show a different behavior, characterized by heavy tails (see Figure S2 and Figure 3 of the main text). These heavy tails of the observed RSAs cannot be captured by a standard negative binomial distribution with $r \in \mathbb{R}^+$. Nevertheless, they can be accommodated when allowing the clustering parameter r to take negative values, $r \in (-1, 0)$, thus enabling us to adapt and generalize the theoretical work of [Tovo et al.(2017)Tovo, Suweis, Formentin, Favretti, Volkov, Banavar et al.] to portray regular statistics of human activities and to use information on local scales to predict hidden features of the human dynamics at the global scale.

We wish now to show that the extension of the parameter region reflects in a power-law behavior of the RSA distribution tail with an exponential cut-off, which well describes the observed patterns in human activities. We point our that both the parameters intervene in the shape of the RSA patterns, being r responsible for the power-law tail with exponent $\alpha = 1 - r$ and ξ for the position of the exponential truncation of the distribution. Note that, although this section is purely theoretical, the predicted exponent $\alpha = 1 - r$ matches very well our findings when we empirically fit the data (see also Figure 3 of the main text).

We start by considering our truncated negative binomial distribution of parameters r and ξ at the global scale (henceforth we will write $P(n)$ for $P(n|1)$, thus omitting the explicit dependence on the scale $p = 1$):

$$P(n) = c(r,\xi)\binom{n+r-1}{n}\xi^n(1-\xi)^r, \tag{6}$$

The following theorem holds true [Walraevens et al.(2012)Walraevens, Demoor, Maertens, and Bruneel, Flajolet and Sedgewick(2008)].

Theorem S1.1 *Let $Y(z)$ be the generating function of a discrete random variable having probability mass function $P(\cdot)$ with dominant singularity R_Y. Let $\beta \in \mathbb{R} \setminus \{0, 1, 2, ...\}$. If for $z \to R_Y$*

$$Y(z) \sim c_Y \left(1 - z/R_Y\right)^{\beta}, \tag{7}$$

then the distribution $P(n)$ satisfies

$$P(n) \sim \frac{c_Y n^{-\beta-1} R_Y^{-n}}{\Gamma(-\beta)} \qquad \text{for } n \to \infty, \tag{8}$$

where $\Gamma(\cdot)$ is the Gamma function.

We wish to apply this theorem to our truncated negative binomial distribution. Let us first recall that a singularity of a complex function is a point in the complex plane where the function is not analytic. Examples are poles, square-root branch points and branch cuts.

We now start by examining the probability generating function:

$$Y(z) = \sum_{n=0}^{\infty} P(n) z^n, \tag{9}$$

where $P(n)$ is given in (6). Observe that, since we wish to investigate the singularities of $Y(z)$, the normalizing factor $c(r, \xi)$ does not play any significant role. Moreover, the tail of a truncated negative binomial is exactly the same of a standard negative binomial, hence we simply disregard of the truncation and conduct the analysis for a standard negative binomial.

Since we aim at finding the lowest-norm singularity of the probability generating function $Y(z)$, we proceed with the computation by replacing the term $P(n)$ in (9) with its definition (6):

$$\begin{aligned} Y(z) &= \sum_{n=0}^{\infty} \binom{n+r-1}{n} \xi^n (1-\xi)^r z^n \\ &= \sum_{n=0}^{\infty} \binom{n+r-1}{n} (z\xi)^n (1-z\xi)^r \cdot \frac{(1-\xi)^r}{(1-z\xi)^r} \\ &= \frac{(1-\xi)^r}{(1-z\xi)^r} \cdot \sum_{n=0}^{\infty} \binom{n+r-1}{n} (z\xi)^n (1-z\xi)^r. \end{aligned}$$

For $z\xi < 1$, i.e. for $z < \frac{1}{\xi}$, the sum converges to 1 as we are summing over $\mathbb{N}$ the marginals of a standard negative binomial of parameters r and $z\xi$.

Thus we are left with

$$Y(z) = \frac{(1-\xi)^r}{(1-z\xi)^r} = c_Y (1 - z\xi)^{-r}.$$

It turns out that $Y(z)$ has a singularity at $z = 1/\xi$.

We now wish to express $Y(z)$ as in (7) to apply the theorem. In our case:

$$Y(z) = c_Y (1 - z\xi)^{-r} = c_Y (1 - z/R_Y)^{\beta},$$

where we set $\beta = -r$ and $R_Y = \frac{1}{\xi}$. Thus, Theorem (S1.1) provides a characterization of the tails of the (truncated) negative binomial:

$$P(n) \sim \frac{c_Y n^{r-1} \xi^n}{\Gamma(-\beta)} = \frac{c_Y n^{r-1} e^{n \ln(\xi)}}{\Gamma(-\beta)}, \qquad n >> 1. \tag{10}$$

S1.5 Estimator for the total number of species and SAC

We proceed now in the description of our procedure. Recall that our method only uses the information available at a sub-sample covering a fraction p^* of the entire system. Therefore, we only have information on the abundances of the S_{p^*} species present within the surveyed area. We now wish to determine the relationship between the total number of species S in the entire population, i.e. at $p = 1$, and the number of observed species at the sub-scale p^*.

Note that the probability that a species among the existing S has null abundance at scale p^* corresponds to the fraction of unsurveyed species. Hence we obtain

$$P(k=0|p^*) \simeq \frac{S - S_{p^*}}{S}. \tag{11}$$

Arranging the latter equation, we get a formula to predict the total number of species:

$$\begin{aligned}
\hat{S} &\overset{\text{eq (11)}}{=} \frac{S_{p^*}}{1 - P(k=0|p^*)} \\
&\overset{\text{eq (2)}}{=} S_{p^*} \frac{1 - (1-\hat{\xi})^{\hat{r}}}{1 - (1-\hat{\xi}_{p^*})^{\hat{r}}} \\
&\overset{\text{eq (4)}}{=} S_{p^*} \frac{1 - \left(1 - \frac{\hat{\xi}_{p^*}}{p^* + \hat{\xi}_{p^*}(1-p^*)}\right)^{\hat{r}}}{1 - (1-\hat{\xi}_{p^*})^{\hat{r}}}.
\end{aligned} \tag{12}$$

Thus we derived a formula to estimate the total number of species of a community given a sample at scale p^*.

Let us note that we can do more. Indeed, for any $q \in (p^*, 1)$ we can apply the same chain of equations as above with some slight modifications to estimate $\hat{S}_q$:

$$\hat{S}_q = S_{p^*} \frac{1 - \left(1 - \frac{\hat{\xi}_{p^*}}{\frac{p^*}{q} + \hat{\xi}_{p^*}(1 - \frac{p^*}{q})}\right)^{\hat{r}}}{1 - (1-\hat{\xi}_{p^*})^{\hat{r}}} = S_{p^*} \frac{1 - \left(\frac{p^*\left(1-\hat{\xi}_{p^*}\right)}{p^* + \hat{\xi}_{p^*}\left(q - p^*\right)}\right)^{\hat{r}}}{1 - (1-\hat{\xi}_{p^*})^{\hat{r}}}. \tag{13}$$

Hence we obtained an explicit formula for the species-accumulation curve for every $q \leq 1$ from the local up to the global scale.

Moreover we can express the RSA distribution at the global scale by plugging the estimated parameters $\hat{\xi}$ and $\hat{r}$ into (1).

S1.6 Popularity and abundance variation through scales

Note that until now we studied the abundance distribution of the observed species at the local scale, but only to estimate the number of unseen species, disregarding of their abundances. However, abundance information may of relevance in some contexts. For example, if one is interested in measuring the popularity of hashtags in Twitter, one naive way to do that is to count the number of times it has been posted. A second novelty we introduced in our work is indeed a method to estimate the variation of popularity in social networks. Let us first recall our previous findings using a more detailed notation which turns out to be essential in the following.

Definition S1.2 *For every $s = 1, ..., S$, we indicate with $n_s^{p^*}$, $n_s^{1-p^*}$ the abundance of species s in the observed (resp. unobserved) fraction p^* (resp. $1 - p^*$) of the population.*

- First, let us introduce the statistics:
$$S_{p^*} = \sum_{s=1}^{S} \mathbb{1}_{\{n_s^{p^*}>0\}}$$
whose expected value can be computed as follows:
$$\mathbb{E}\left[S_{p^*}\right] = \mathbb{E}\left[\sum_{s=1}^{S} \mathbb{1}_{\{n_s^{p^*}>0\}}\right] = \sum_{s=1}^{S} \mathbb{E}\left[\mathbb{1}_{\{n_s^{p^*}>0\}}\right] = \sum_{s=1}^{S} \mathbb{P}\left(n_s^{p^*}>0\right)$$
$$= S \cdot P\left(k>0|p^*\right) = S \cdot \left[1 - P\left(k=0|p^*\right)\right].$$
- Arranging the latter equation, we can isolate the quantity we are interested to estimate:
$$S = \frac{\mathbb{E}\left[S_{p^*}\right]}{1 - P\left(k=0|p^*\right)}. \tag{14}$$
- An estimator of S can be thus obtained by replacing the mean $\mathbb{E}\left[S_{p^*}\right]$ by the observable S_{p^*}:
$$\hat{S} = \frac{S_{p^*}}{1 - P\left(k=0|p^*\right)} \tag{15}$$
With no surprise, we recover the same result as in (12). We wish to stress that this new formulation allows us to push further our investigation, as we are going to show.

We wish now to apply the same procedure to different statistics.
Recall that we are sampling S_{p^*} species at scale p^* from a pool consisting of N individuals belonging to S different species. If a species s is not observed in the sample at scale p^*, we say that s is a "new" species. The meaning of this definition can be easily explained. If you imagine to further sample your population, you can either pick individuals belonging to species already observed or you can discover indeed "new" species.

Let us then consider the following statistics for the new species:
$$S_{1-p^*}^{\text{new}} = \sum_{s=1}^{S} \mathbb{1}_{\{n_s^{p^*}=0, n_s^{1-p^*}>0\}}. \tag{16}$$
The following chain of equality turns out to be meaningful in the following:
$$S_{1-p^*}^{\text{new}} = \sum_{s=1}^{S} \mathbb{1}_{\{n_s^{p^*}=0, n_s^{1-p^*}>0\}} = \sum_{s=1}^{S} \mathbb{1}_{\{n_s^{p^*}=0, n_s^{1}>0\}}$$
$$= \sum_{s=1}^{S} \mathbb{1}_{\{n_s^{p^*}=0\}} = \sum_{s=1}^{S} \left(1 - \mathbb{1}_{\{n_s^{p^*}>0\}}\right) = S - S_{p^*}.$$
We can recover an estimator for the "new" species from estimator (15) for S.

This remark seems trivial, and the chain of equation above appears redundant. Nevertheless, it is crucial for the development of our work. We stress that the statistics $S_{1-p^*}^{\text{new}}$ uses both the information at the sample scale p^* and the information contained

in the unseen fraction of the population $1-p^*$. In contrast, the statistics for S_{p^*} only consider the observed individuals.

Given now the statistics (16) representing the number of species unobserved in the sample of size p^*N but present in the remaining population of size $(1-p^*)N$. We wish to recover an estimator for the new species $S_{1-p^*}^{\text{new}}$. We thus compute the expected value of the corresponding statistics:

$$\mathbb{E}\left[S_{1-p^*}^{\text{new}}\right] = \mathbb{E}\sum_{s=1}^{S} \mathbb{1}_{\{n_s^{p^*}=0, n_s^{1-p^*}>0\}} = S\cdot\mathbb{P}\left(n_s^{p^*}=0, n_s^{1-p^*}>0\right)$$
$$= S\cdot\mathbb{P}\left(n_s^{p^*}=0, n_s^{1}>0\right) = S\cdot\underbrace{\mathbb{P}\left(n_s^{p^*}=0\right)}_{P\left(k=0|p^*\right)}.$$

The expected value turns out to be a product of two factors: $P\left(k=0|p^*\right) = \mathbb{P}(n_s^{p^*}=0)$, which can be computed via (2), and S, a quantity we can estimate via (15). Hence we derive the following estimator:

$$\hat{S}_{1-p^*}^{\text{new}} = \frac{S_{p^*}}{1-P\left(k=0|p^*\right)}\cdot P\left(k=0|p^*\right).$$

This procedure captures the techniques which allows us to derive other useful estimators.

In particular, this turning point leads us to new statistics that consider also the popularity.
Let us start from the statistics:

$$S_{1-p^*}^{\text{new}}(l) = \sum_{s=1}^{S} \mathbb{1}_{\{n_s^{p^*}=0, n_s^{1-p^*}=l\}}. \tag{17}$$

Note that if we get an expression for $S_{1-p^*}^{\text{new}}(l)$, than we can easily extend the result to

$$S_{1-p^*}^{\text{new}}(\geq L) = \sum_{l=L}^{\cdot} S_{1-p^*}^{\text{new}}(l).$$

Moreover, results from the previous section can be included here, simply noticing that:

$$S_{1-p^*}^{\text{new}} = S_{1-p}^{\text{new}}(\geq 1) = \sum_{l=1}^{\cdot} S_{1-p^*}^{\text{new}}(l).$$

We proceed as before by computing the expected value:

$$\begin{aligned}
\mathbb{E}\left[S_{1-p^*}^{\text{new}}(l)\right] &= \mathbb{E}\left[\textstyle\sum_{s=1}^{S} \mathbb{1}_{\{n_s^{p^*}=0, n_s^{1-p^*}=l\}}\right] \\
&= S\cdot\mathbb{P}\left(n_s^{p^*}=0, n_s^{1-p^*}=l\right) \\
&= S\cdot\mathbb{P}\left(n_s^{p^*}=0, n_s^{1}=l\right) \\
&= S\cdot\underbrace{\mathbb{P}\left(n_s^{p^*}=0|n_s^{1}=l\right)}_{Binomial(n_s^1, p^*)}\underbrace{\mathbb{P}\left(n_s^{1}=l\right)}_{P\left(l|1\right)},
\end{aligned}$$

where in the third equality we used the following relation:

$$\mathbb{P}\left(n_s^{p^*}=x, n_s^{1-p^*}=y\right) = \mathbb{P}\left(n_s^{p^*}=x, n_s^{1}=x+y\right).$$

Let us note now the following facts:

- From the sampling binomial distribution, it holds that $\mathbb{P}\left(n_s^{p^*} = 0 | n_s^1 = l\right) = (1-p^*)^l$;
- $\mathbb{P}\left(n_s^1 = l\right) = P(l|1)$ is given by (1);
- S is unknown and we thus need an estimator for it.

Again, we can use the results of the previous subsection to define $\hat{S} = \dfrac{S_{p^*}}{1 - P\left(k=0|p^*\right)}$ and hence to obtain

$$\hat{S}^{\text{new}}_{1-p^*}(l) = \hat{S} \cdot (1-p^*)^l \cdot P(l|1) = \frac{S_{p^*}}{1 - P\left(k=0|p^*\right)} \cdot (1-p^*)^l \cdot P(l|1), \tag{18}$$

which is the estimator for the new species with abundance l.

Thus, as a first partial result, we obtained an estimator for the popularity of the new species.

Let us now consider the statistics:

$$S_{1-p^*}(l \to k) = \sum_{s=1}^{S} \mathbb{1}_{\{n_s^{p^*}=l, n_s^{1-p^*}=k\}}, \tag{19}$$

which represents the number of species having contemporarily abundance l at the observed scale p^* and abundance k at the unobserved scale $1-p^*$. Note that we can compute the number of species having an abundance that lies within a population interval by summing up on different values of l and k. We proceed by computing the expected value of the statistics (19):

$$\begin{aligned}
\mathbb{E}\left[S_{1-p^*}(l \to k)\right] &= \mathbb{E}\left[\textstyle\sum_{s=1}^{S} \mathbb{1}_{\{n_s^{p^*}=l, n_s^{1-p^*}=k\}}\right] \\
&= S \cdot \mathbb{P}\left(n_s^{p^*} = l, n_s^{1-p^*} = k\right) \\
&= S \cdot \mathbb{P}\left(n_s^{p^*} = l, n_s^{1} = k+l\right) \\
&= S \cdot \underbrace{\mathbb{P}\left(n_s^{p^*} = l | n_s^{1} = k+l\right)}_{Binomial(n_s^1, p^*)} \underbrace{\mathbb{P}\left(n_s^{1} = k+l\right)}_{P(k+l|1)}.
\end{aligned}$$

Now we have the following:

- From the sampling binomial distribution, it holds that $\mathbb{P}\left(n_s^{p^*} = l | n_s^1 = k+l\right) = \binom{k+l}{l} p^{*l}(1-p^*)^k$;
- $\mathbb{P}\left(n_s^1 = k+l\right) = P(k+l|1) = c(r,\xi)\binom{k+l+r-1}{k+l}\xi^{k+l}(1-\xi)^r$;
- S is unknown. However, we can estimate it via $\hat{S} = \dfrac{S_{p^*}}{1 - P\left(k=0|p^*\right)}$.

Hence we obtained

$$\begin{aligned}
\hat{S}_{1-p^*}(l \to k) &= \hat{S} \cdot \mathbb{P}\left(n_s^{p^*} = l | n_s^1 = k+l\right) \cdot P(k+l|1) \\
&= \frac{S_{p^*}}{1 - P(0|p^*)} \cdot \binom{k+l}{l} p^{*l}(1-p^*)^k \cdot c(r,\hat{\xi})\binom{k+l+\hat{r}-1}{k+l}\hat{\xi}^{k+l}(1-\hat{\xi})^{\hat{r}}.
\end{aligned}$$

Estimator $\hat{S}_{1-p^*}(l \to k)$ above gives the number of species with abundance l at the observed scale p^* and abundance k at the unobserved scale $1-p^*$. Note that this estimator is independent of the number of species with abundance l at scale p^*; indeed,

we are using the sample at scale p^* only to estimate the parameters ξ_{p^*} and r, which we need to predict $\hat{S}$. Hence we are only using partial information at the local scale. We wish now to take into account the information about the number of species with abundance l at the surveyed scale, $S_{p^*}(l)$. In particular, we are looking for an estimator of the species with abundance k in the unobserved fraction $1-p^*$ of the population, given that they have abundance l in the sample at the observed scale p^*.

We thus define $S_{p^*}(l) := \sum_{s=1}^{S} \mathbb{1}_{\{n_s^{p^*}=l\}}$.

In the following we will need to use quantities of the type $\mathbb{P}(n_s^{1-p^*}=k|n_s^{p^*}=l)$.

Using Bayes' theorem, we obtain

$$\begin{aligned}\mathbb{P}(n_s^{1-p^*}=k|n_s^{p^*}=l) &= \mathbb{P}(n_s^1-n_s^{p^*}=k|n_s^{p^*}=l)\\ &= \mathbb{P}(n_s^1-l=k|n_s^{p^*}=l)\\ &= \mathbb{P}(n_s^1=k+l|n_s^{p^*}=l)\\ &= \frac{\mathbb{P}(n_s^{p^*}=l|n_s^1=k+l)\mathbb{P}(n_s^1=k+l)}{\mathbb{P}(n_s^{p^*}=l)}.\end{aligned}$$

Note that we all the probabilities appearing in the latter formula are known, since:

- $\mathbb{P}\left(n_s^{p^*}=l|n_s^1=k+l\right)=\binom{k+l}{l}p^{*l}(1-p^*)^k$ is the sampling binomial distribution;
- $\mathbb{P}\left(n_s^1=k+l\right)=P(k+l|1)=c(r,\xi)\binom{k+l+r-1}{k+l}\xi^{k+l}(1-\xi)^r$ is the global truncated negative binomial distribution of parameters r and ξ as in (1);
- $\mathbb{P}\left(n_s^{p^*}=l\right)=P(l|p^*)=c(r,\xi)\binom{l+r-1}{l}\xi_p^{*l}(1-\xi_{p^*})^r$ is again a truncated negative binomial with rescaled parameter ξ_p as in (2).

Let us now retrace the same steps as for $\hat{S}_{1-p^*}(l\to k)$ for the conditional estimator $\hat{S}_{1-p^*}(k|l)$. We start from the statistics

$$S_{1-p^*}(k|l)=\sum_{s=1}^{S}\mathbb{1}_{\{n_s^{p^*}=l\}}\mathbb{1}_{\{n_s^{1-p^*}=k,n_s^{p^*}=l\}}=\sum_{s=1}^{S_{p^*}(l)}\mathbb{1}_{\{n_s^{1-p^*}=k|n_s^{p^*}=l\}}.$$

We proceed by computing the expected value

$$\mathbb{E}\left[S_{1-p^*}(k|l)\right]=S_{p^*}(l)\cdot\mathbb{P}\left(n_s^{1-p^*}=k|n_s^{p^*}=l\right)=S_{p^*}(l)\cdot\frac{\mathbb{P}\left(n_s^{p^*}=l|n_s^1=k+l\right)\mathbb{P}\left(n_s^1=k+l\right)}{\mathbb{P}\left(n_s^{p^*}=l\right)}.$$

Note that empirically $\mathbb{P}\left(n_s^{p^*}=l\right)=S_{p^*}(l)/S$, so that we can recover $\mathbb{E}\left[S_{1-p^*}(l\to k)\right]$.

Let us now insert into the above formula the probabilities computed by using the fitted parameters:

$$\hat{S}_{1-p^*}(k|l)=S_{p^*}(l)\cdot\frac{\binom{k+l}{l}p^{*l}(1-p^*)^k\cdot\binom{k+l+\hat{r}-1}{k+l}\hat{\xi}^{k+l}(1-\hat{\xi})^{\hat{r}}}{\binom{l+\hat{r}-1}{l}\hat{\xi}_{p^*}^l(1-\hat{\xi}_{p^*})^{\hat{r}}},$$

where the terms $c(r,\hat{\xi})$ in the numerator has cancelled out with the one at the denominator.

Estimator $\hat{S}_{1-p}(k|l)$ is theoretically unbiased.

Note that, again, we can pass from punctual estimation to cumulative ones, by summing up over all l and k values above some fixed thresholds L and K, respectively:

$$\hat{S}_{1-p^*}(\geq K| \geq L) = \sum_{l \geq L} \sum_{k \geq K} \hat{S}_{1-p^*}(k|l) \tag{20}$$

Estimator (20) is the one we are going to test in our databases.

S2 Additional results and figures

In this section we collect some additional results not presented in the main text.

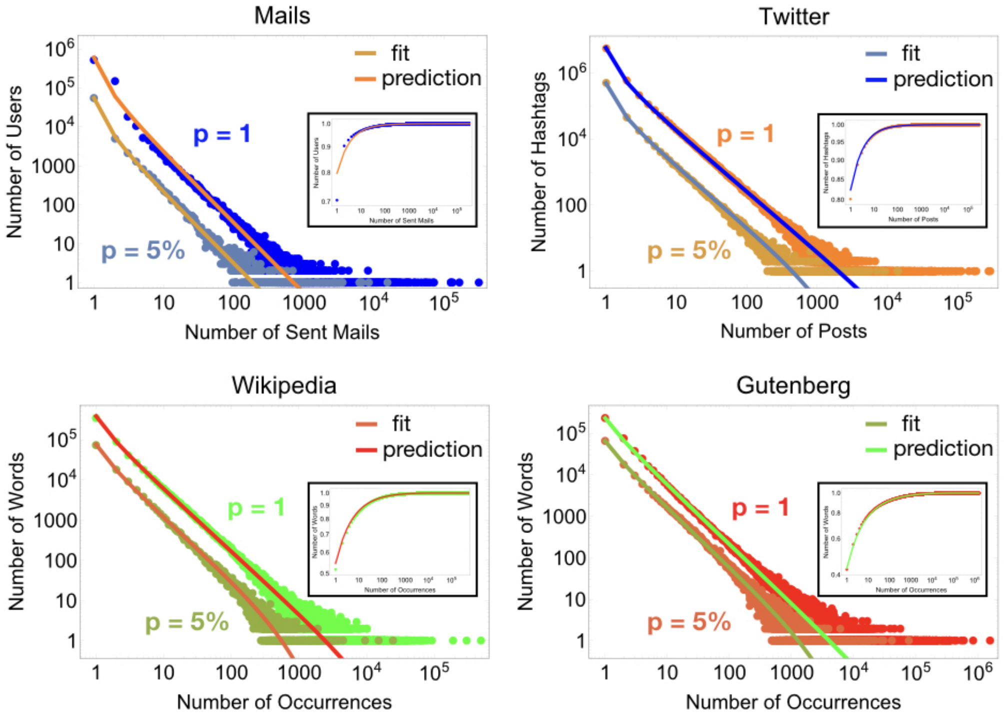

Figure S1. Best-fit and predicted patterns from a local sample scale $\mathbf{p^* = 5\%}$. Empirical RSA curves at global scale ($p = 1$) and local scale ($p^* = 5\%$) are shown. In each panel, coloured lines over the local RSAs represent the distribution obtained via a best-fit of the empirical pattern with a negative binomial having $r \in (-1, 0)$. Lines over the global RSA distributions represent our prediction for the RSAs at the global scales obtained via our upscaling equations for both the parameters and the biodiversity. In each panel, insets showing the corresponding global cumulative RSA (both empirical and predicted) are added.

S2.1 Upscaling results from sample scale $p^* = 3\%$

In the main text we showed the results we obtained with our upscaling method when sampling a fraction $p^* = 5\%$ of the four databases. We performed the same tests also for a local scale $p^* = 3\%$, with similar results.

First of all, as shown in Figure S2, also for the case $p^* = 3\%$ we observe the form-invariance property of the empirical RSAs for all the considered human activity datasets.

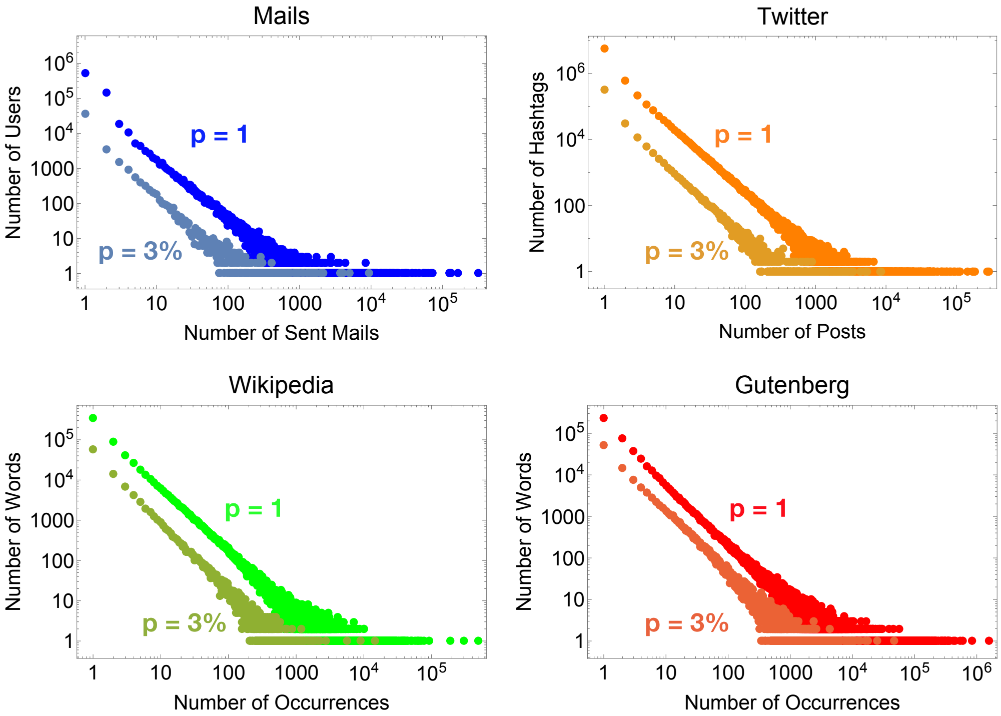

Figure S2. Universality and form-invariance of the empirical RSAs. Empirical RSA curves at the global scale ($p = 1$) and the local scale ($p^* = 3\%$) are shown. RSA is scale-free in all the four datasets analyzed, with a power-law form maintained through the different human activities and scales. RSA form-invariance property is at the core of our theoretical framework.

Moreover, as for $p^* = 5\%$, we tested the reliability of estimator (12) in predicting the total number of species in the different networks when only a random portion of them is extracted. Table S3 displays the relative percentage error we obtained for the different databases together with the total dataset composition and the values of the parameters fitted from the empirical RSAs at $p^* = 3\%$.

S2.2 Upscaling results for popularity change

In the main text we exhibited in Table 2 the results for the predictions of popularity (via the conditional estimator 20) in the unsurveyed fraction $1 - p^* = 0.95$ of the population for a fixed value of the local popularity threshold $L = 10$. In Table S4 we show the results obtained for different values of L and K.

S2.3 Local Analysis

We also tested how estimator (12) performs on different spatial sub-scales. In this case, due to the huge amount of data, we chose to work with a smaller datasets for a systematic analysis. In particular, we considered as global four samples of the original datasets each covering a fraction $p^* = 5\%$ of the total amount of data (see Figure S3).

We then randomly sub-sampled the reduced 5% databases at different sub-scales p^{**} ranging from 10% to 90% and applied our framework to predict the number of species observed at p^* (here considered as $p = 1$).

In Figure S3, bottom panels, we displayed the relative percentage error graphs

	Emails	Twitter	Wikipedia	Gutenberg
Species	$752,299$	$6,972,453$	$673,872$	$554,193$
Individuals	$6,914,872$	$34,696,973$	$29,606,116$	$126,289,661$
$\mathbf{r}$	-0.788	-0.828	-0.549	-0.422
$\xi_{\mathbf{p}^*}$	0.9997	0.9976	0.9987	0.9994
Relative Error	-2.74%	4.41%	8.22%	-3.52%

Table S3. Predicted relative errors. Upscaling results for the number of species of the four analysed datasets from a local sample covering a fraction $p^* = 3\%$ of the global database. For each database, we display the number of species (users, hashtags, words) and individuals (sent mails, posts, occurrences) at the global scale, together with the fitted RSA distribution parameters at the sampled scale and the relative percentage error between the true number of species and the one predicted by our framework.

Table S4. Percentage errors for popularity change predictions in Twitter database. For $L = 10,\ 40,\ 55$ (first column) and different values of K(second column), we estimated, from ten different Twitter samples (at $p = 5\%$), the number of species having abundance at least K at the unobserved scale $1 - p^* = 95\%$ given that they have abundance at least L at the sampled scale p^* (see estimator 4 of the main text). The average true number of species $S_{1-p^*}(\geq K| \geq L)$ and the average one predicted by our method among the ten sub-samples are displayed in the third and fourth columns, respectively. Finally, in the last two columns, we inserted the mean and the variance of the relative error obtained among the ten predictions.

L	K	$S_{1-p^*}(\geq K\| \geq L)$	$\hat{S}_{1-p^*}(\geq K\| \geq L)$	Relative Error	Variance
10	77	$14,266$	$14,274.38$	-0.0029	0.0012
10	115	$14,113$	$14,105.65$	0.0534	0.0151
10	154	$13,551$	$13,544.76$	0.2457	0.0428
10	192	$12,509$	$12,584.32$	0.4679	0.0731
10	231	$11,305$	$11,366.66$	0.5372	0.0965
40	362	$3,749$	$3,748.99$	-0.0001	≈ 0
40	543	$3,742$	$3,741.96$	0.0393	0.0058
40	724	$3,591$	$3,578.83$	-0.0715	0.0668
40	905	$3,096$	$3,091.45$	0.0368	0.0660
40	$1,086$	$2,600$	$2,582.75$	-0.5634	0.0370
55	504	$2,673$	$2,673.00$	≈ 0	≈ 0
55	756	$2,672$	$2,670.96$	-0.0141	0.0013
55	$1,008$	$2,569$	$2,567.71$	-0.0978	0.0565
55	$1,260$	$2,195$	$2,199.11$	0.0023	0.0557
55	$1,512$	$1,806$	$1,820.01$	0.1286	0.2070

between the true number of species, S^*, and the one predicted from the local information at the different sub-scales p^{**}. We see that, for all datasets and sub-scales, our method always led to an error below 5%. Moreover, it displays an intuitive decreasing behavior as the available information increases, a desirable property for an estimator. We performed the same analysis also starting from a sample at the scale $p^* = 3\%$, obtaining comparable results (see Figure S4).

Figure S3. Relative percentage errors at different sub-scales from $\mathbf{p^* = 5\%}$. Starting from a sample at $p^* = 5\%$ of each human activity database, we sub-sampled it at different spatial sub-scales $p^{**} \in \{10\%, \ldots, 90\%\}$ of p^* and computed the relative percentage error between the number of predicted species, $\hat{S}^*$, and the true number of species, S^*, observed in the sample at p^*, here considered as the global scale ($p^* = 1$).

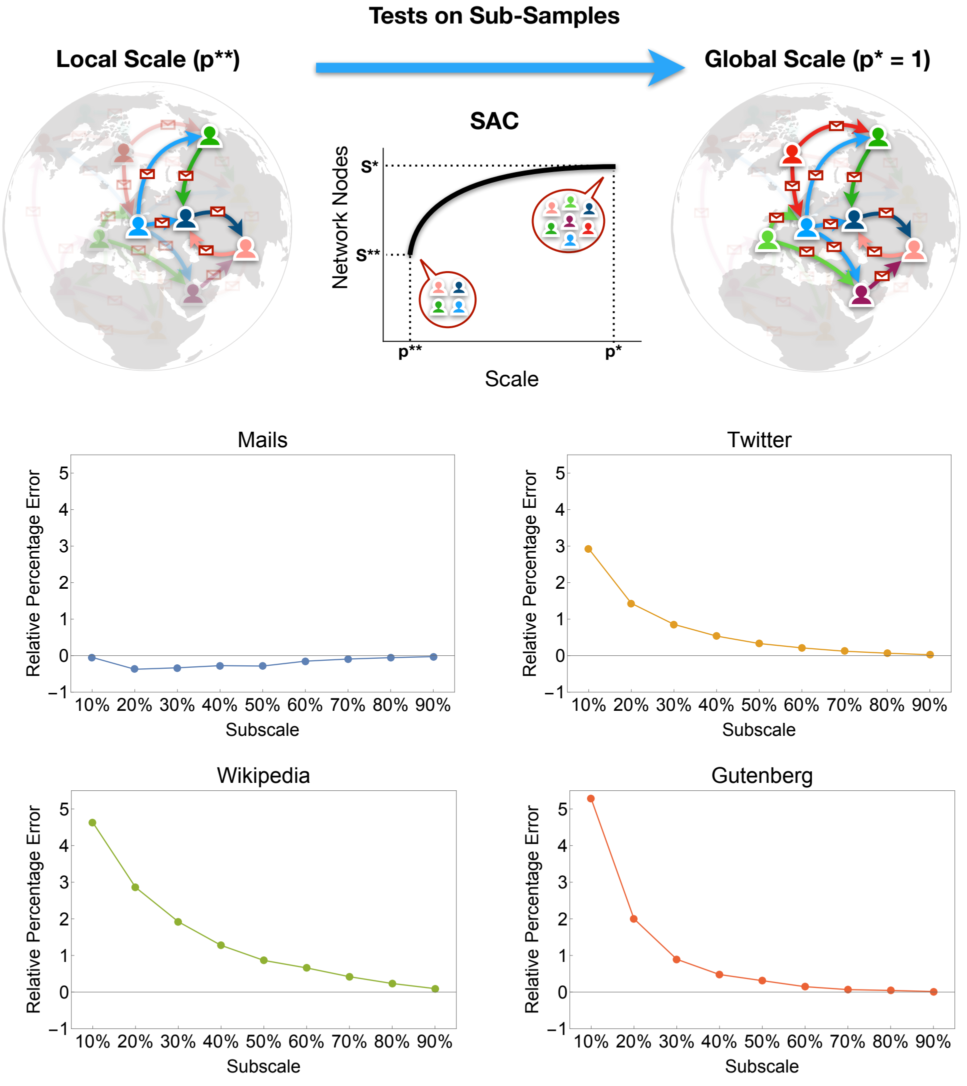

Figure S4. Relative percentage errors at different sub-scales from $\mathbf{p}^* = 3\%$. Starting from a sample at $p^* = 3\%$ of each human activity database, we sub-sampled it at different spatial sub-scales $p^{**} \in \{10\%, \ldots, 90\%\}$ of p^* and computed the relative percentage error between the number of predicted species, $\hat{S}^*$, and the true number of species, S^*, observed in the sample at p^*, here considered as the global scale ($p^* = 1$).

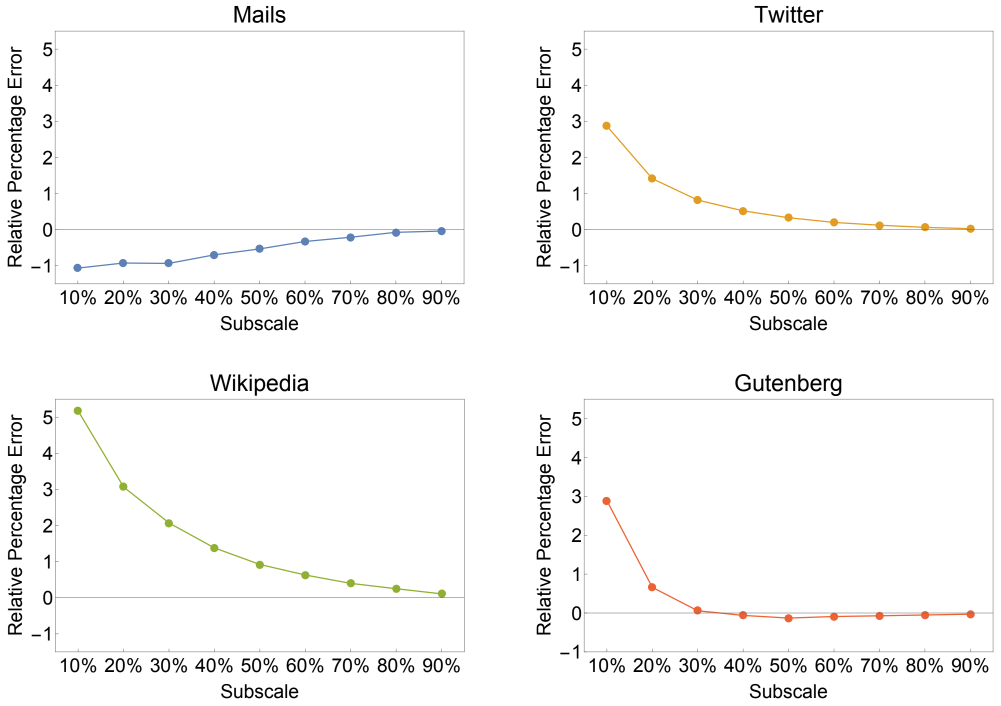

References

Flajolet and Sedgewick(2008). Flajolet, P. and Sedgewick, R. (2008). *Analytic Combinatorics* (Cambridge University Press)

Tovo et al.(2019)Tovo, Formentin, Suweis, Stivanello, Azaele, and Maritan. Tovo, A., Formentin, M., Suweis, S., Stivanello, S., Azaele, S., and Maritan, A. (2019). Inferring macro-ecological patterns from local species' occurrences. *Oikos*

Tovo et al.(2017)Tovo, Suweis, Formentin, Favretti, Volkov, Banavar et al.. Tovo, A., Suweis, S., Formentin, M., Favretti, M., Volkov, I., Banavar, J. R., et al. (2017). Upscaling species richness and abundances in tropical forests. *Science advances* 3, e1701438

Walraevens et al.(2012)Walraevens, Demoor, Maertens, and Bruneel. Walraevens, J., Demoor, T., Maertens, T., and Bruneel, H. (2012). Stochastic queueing-theory approach to human dynamics. *Physical Review E* 85, 021139
